# Supplementary material for: Detection of false position attacks in VANETs through bagging ensemble learning
Source: PLoS One. 2025 Aug 1;20(8):e0328829. doi: 10.1371/journal.pone.0328829 (PMC12316297; doi:10.1371/journal.pone.0328829)
Supplement: Attachment — Submitted filename: Response_to_Reviewers_PONE-D-25-17735.docx [file pone.0328829.s002.docx]

**Response to Reviewers Comments**

Journal: **PLOS ONE**

Manuscript ID: **PONE-D-25-17735**

Manuscript Title: **Detection of False Position Attacks in VANETs through Bagging Ensemble Learning**

**Reviewer #1:**

1. The experiments are solely based on VeReMi simulation data and do not validate performance in real-world traffic scenarios (e.g., urban congestion, high-speed mobility). Could real-road data testing or comparisons with public VANET real-world datasets (e.g., CarTel) be added to verify the generalization capability of the proposed model?

**Author Response:** We acknowledge the reviewer’s concern regarding the need to evaluate the model's generalization beyond the VeReMi simulation dataset. While publicly available real-world VANET datasets such as CarTel or NGSIM provide valuable vehicle trajectory data, they unfortunately do not include labeled position falsification attacks. As such, these datasets cannot be directly used for supervised attack detection evaluation without synthetic manipulation.

To address this limitation, we conducted additional experiments using the VeReMi dataset by **simulating realistic traffic conditions**. Specifically, we categorized the data into **urban** (vehicle speed < 10 km/h) and **highway** (vehicle speed > 70 km/h) scenarios, representing urban congestion and high-speed mobility, respectively. The model was then evaluated across all available attack types (Type-1, 2, 4, 8, and 16) within each traffic condition. The results, presented in Table 6, demonstrate consistently high accuracy, precision, and F1-scores across these diverse mobility scenarios.

2. The experiments only cover five predefined attack types (e.g., fixed-position and random-offset attacks) and do not address more stealthy attacks (e.g., dynamic-offset or coordinated attacks). Due to insufficient coverage of attack types, could the attack scenarios be expanded—for instance, by simulating multi-node coordinated position spoofing or dynamically adjusted attack strategies—to evaluate the model’s capability in handling unknown attacks?

**Author Response:** We thank the reviewer for highlighting this important aspect of attack diversity. In our current study, we have evaluated the model using five attack types from the VeReMi dataset (Types 1, 2, 4, 8, and 16), each representing different falsification strategies:

- **Type-1 (Constant Position)** and **Type-2 (Constant Offset)** reflect simple static attacks.
- **Type-4 (Random Position)** and **Type-8 (Random Offset)** introduce unpredictable falsification.
- **Type-16 (Eventual Stop)** simulates stealthier behavior where a vehicle appears immobilized temporarily.

While this taxonomy includes both basic and moderately deceptive tactics, we acknowledge that more **sophisticated threats**, such as **dynamic-offset** attacks and **multi-node coordinated spoofing**, remain unexplored in the current work. To partially address this gap, we have included **Type-16**, which already captures a **subtle temporal deception**. However, the VeReMi dataset does not natively support coordinated or adaptive attack scenarios.

3. The "black-box" nature of KNN+Bagging may affect trustworthiness in practical deployment, and the paper lacks analysis on the contributions of critical features. It is recommended to further supplement the analysis in this aspect.

**Author Response:** We thank the reviewer for highlighting the need for improved interpretability. To address this, we added a new subsection (Section 4.3.1, "**Feature Importance Analysis**"), presenting a permutation importance analysis for the features used (pos-x1, pos-y1, spd-x1, spd-y1, pos-x2, pos-y2, spd-x2, spd-y2, time_interval). The results, shown in Table 3 identify pos-x2 and pos-y2 as the most critical features, with scores up to 0.3732 and 0.3728 for Attack 4 and Attack 8, respectively, reflecting the dataset’s positional attack patterns. The zero importance of time_interval suggests limited temporal variability, likely due to fixed BSM intervals.

4. The experimental comparisons are limited to traditional machine learning models and do not include some latest advanced methods, such as Graph Neural Networks (GNN) and Federated Learning (FL). Could more comparative experiments or references to recent literature analyses be added to verify the superiority of the proposed method?

**Author Response:** While GNNs and FL are promising approaches for misbehavior detection in VANETs, their integration with bagging is non-trivial and introduces significant computational and architectural overhead. Our proposed bagging-based ensemble was chosen for its practical suitability in RSU-level real-time deployment, simplicity, and strong performance on simulated spatial attack scenarios. Future work may explore hybrid models combining GNNs or FL with ensemble learning to handle more complex or decentralized environments.

5. The study does not consider the impact of practical constraints in real-world deployment, such as communication interruptions and network latency between RSUs (Roadside Units) and vehicles, on the proposed detection system. It is suggested that the authors discuss this aspect.

**Author Response:** We thank the reviewer for emphasizing real-world deployment constraints. Our original manuscript indirectly referenced delays (Section 1) and real-world validation (Section 7) but lacked a specific discussion of RSU-vehicle communication issues. We have addressed this by adding a paragraph in Section 6 (Discussion); see the last paragraph.

**Reviewer #2:**

This paper proposes an ensemble learning framework that uses CART, Random Forest, KNN, and MLP classifiers with bagging for Vehicular Ad-hoc Networks vulnerable to location forgery attacks to detect such attacks. By analyzing sequential BSMs from the VeReMi dataset via an RSU-level system, they get the results that KNN with bagging achieved 100% precision, recall, accuracy, and F1 score for Attack 1 and near-perfect performance for complex attacks. Other models have varying performance across simple and complex scenarios. The framework proves the effectiveness of ensemble methods, particularly KNN with bagging, in securing VANETs. The following questions are discussed with the author.

1. It is recommended to introduce the Public Key Infrastructure (PKI) model in the Introduction part before explaining its advantages and functions relative to Intelligent Transportation Systems (ITS).

**Author Response:** We agree with the reviewer’s suggestion and have revised the Introduction accordingly. The explanation of the Public Key Infrastructure (PKI) model has been added to the first paragraph of the Section 1 (Introduction).

2. The connection between the first and second paragraphs of the article is not strong. It is recommended to add content to smoothly connect the first paragraph, which emphasizes the necessity of a bad behavior detection model, with the content in the second paragraph regarding the security threats faced by Vehicular Ad Hoc Networks (VANETs).

**Author Response:** We thank the reviewer for highlighting the need for a smoother transition between the first and second paragraphs of the Introduction. To address this, we have revised the end of the first paragraph to better connect with the discussion on security threats in VANETs presented in the second paragraph. This adjustment improves the coherence and logical flow of the Introduction section.

3. The Introduction part and the Rationale part of the article have introduced the methods used in this article many times. The order of the methods lacks a logical connection, the transition is rigid, and a clear theoretical system is not formed. To enhance the readability and academic standardization of the article, it is suggested that the author should reorganize the chapter structure of the method, integrate the repetitive content, adopt the method of comparative analysis or progressive logic arrangement, simplify the expression, and highlight the core technical details and innovation points.

**Author Response:** We appreciate the reviewer’s feedback on the repetitive method introductions and lack of logical flow in the Introduction and Rationale sections. To address this, we revised Section 1 (Introduction) and Section 2 (Rationale) as suggested.

4. It is recommended to introduce the existing applications of the four classification models used in this study, such as Decision tree, Random Forest, K-Nearest Neighbor algorithm and Multilayer Perceptron classifier, so that readers can better understand the research mechanism of this study.

**Author Response:** We thank the reviewer for recommending the inclusion of existing applications of the classification models used in this study. In response, we have revised Section 4.4 (Classification Models Used) to include brief overviews of typical applications for each model. Additionally, we have added Table 4: Comparative Analysis of Bagging-Enhanced Classification Models, which summarizes the characteristics of each model.

5. Because the method proposed in this paper aims to further improve the accuracy and robustness of detection, add the following research question to solve the problem of bad behavior detection in vehicular ad hoc networks (VANETs): "How does this study further improve the robustness of detection?" And add the relevant content in the article to explain it.

**Author Response:** We thank the reviewer for highlighting the need to emphasize robustness. We added the new research question, as suggested, in Section 3 (Research Questions). To explain this, we revised Section 4.5 by expanding the first paragraph to detail how robustness is enhanced.

6. It is recommended to add a comparative analysis of the four machine learning models combined with the bagging method in the fourth part of the article, presented in the form of tables, so that their respective application scenarios, similarities and differences are more intuitive.

**Author Response:** We thank the reviewer for recommending a comparative analysis to clarify the roles and distinctions of the machine learning models used in our study. In response, we have revised Section 4.4 to include a more detailed discussion and added Table 4, which presents a comparative analysis of the four models combined with the bagging method. The table highlights their respective application scenarios, similarities, and differences to provide a more intuitive understanding for readers.

7. In this paper, the bagging method is combined with four machine learning models, but only the hyperparameter tuning of KNN is performed. The author explains the reason for choosing KNN in the paper.

**Author Response:** We thank the reviewer for noting our focus on hyperparameter tuning for the K-Nearest Neighbors (KNN) model. To clarify this choice, we have added a justification in the last paragraph of Section 4.5, explaining the rationale for prioritizing KNN and its relevance to our study's objectives.

8. In this paper, the selection of hyperparameters is based on the preliminary experimental results. However, to make readers better understand the basis and rationality of the selection of hyperparameters, it is suggested that the author should increase the principle and process of the preliminary experiment, so that other researchers can refer to and learn from similar work.

**Author Response:** We thank the reviewer for suggesting more details on the preliminary experiments for hyperparameter selection. We have addressed this in the 2^nd^ paragraph of Section 4.5.

9. In the research, although accuracy, recall, precision and F1-score are proposed as the evaluation indexes of the generalization ability of the model, the specific data of accuracy, recall, precision and F1 - score are not elaborated and analyzed in the text, which leads to the absence of key data in the evaluation system and affects the integrity and objectivity of the model performance evaluation.

**Author Response:** Thank you for the valuable feedback. We have addressed this concern by adding a new subsection (Section 5.5) that provides a detailed comparative analysis of the models using accuracy, precision, recall, and F1-score. Table 5 already includes the specific values of these metrics for each classifier and attack type. In the revised manuscript, we now explicitly elaborate on and interpret these results to ensure a comprehensive and objective evaluation of model performance.

**We would like to express our gratitude for your valuable feedback. We have addressed each of your comments and made the necessary revisions to improve the manuscript.**
